# The effect of occupational exposure to welding fumes on trachea, bronchus and lung cancer: A protocol for a systematic review and meta-analysis from the WHO/ILO Joint Estimates of the Work-related Burden of Disease and Injury

**DOI:** 10.1016/j.envint.2020.106089

**Published:** 2020-12

**Authors:** Frank Pega, Nicholas Chartres, Neela Guha, Alberto Modenese, Rebecca L. Morgan, Martha S. Martínez-Silveira, Dana Loomis

**Affiliations:** a Department of Environment, Climate Change and Health, World Health Organization, Geneva, Switzerland; b Program on Reproductive Health and the Environment, University of California, San Francisco, San Francisco, CA, United States; c Office of Environmental Health Hazard Assessment, California Environmental Protection Agency, Oakland, CA, United States; d Department of Biomedical, Metabolic and Neural Sciences, University of Modena & Reggio Emilia, Modena, Italy; e Department of Health Research Methods, Evidence, and Impact, McMaster University, Hamilton, Canada; f Gonçalo Moniz Institute, Oswaldo Cruz Foundation, Candeal-Salvador, BA, Brazil; g School of Community Health Sciences, University of Nevada, Reno, Reno, NV, United States

**Keywords:** Global burden of disease, Occupational health, Protocol, Systematic review, Meta-analysis, Welding, Tracheal neoplasms, Lung neoplasms

## Abstract

**Background:**

The World Health Organization (WHO) and the International Labour Organization (ILO) are developing joint estimates of the work-related burden of disease and injury (WHO/ILO Joint Estimates), with contributions from a large network of experts. Welding fumes have been classified as carcinogenic to humans (Group 1) by the International Agency for Research on Cancer (IARC); this assessment found sufficient evidence from studies in humans that welding fumes are a cause of lung cancer. In this article, we present the protocol for a systematic review of parameters for estimating the number of deaths and disability-adjusted life years from trachea, bronchus and lung cancer attributable to occupational exposure to welding fumes, to inform the development of the WHO/ILO Joint Estimates.

**Objectives:**

We aim to systematically review and meta-analyse estimates of the effect of occupational exposure to welding fumes on trachea, bronchus and lung cancer, applying the Navigation Guide systematic review methodology as an organizing framework.

**Data sources:**

We will search electronic bibliographic databases for potentially relevant records from published and unpublished studies, including Medline, EMBASE, Web of Science, and CISDOC. We will also search electronic grey literature databases, Internet search engines and organizational websites; hand search reference list of previous systematic reviews and included study records; and consult additional experts.

**Study eligibility and criteria:**

We will include working-age (≥15 years) workers in the formal and informal economy in any Member State of WHO and/or ILO but exclude children (<15 years) and unpaid domestic workers. The eligible risk factor will be occupational exposure to welding fumes, measured directly or indirectly (i.e., through proxy of relevant occupation, work task, job-exposure matrix, expert judgment or self-report). The eligible outcomes will be trachea, bronchus and lung cancer. We will include randomized controlled trials, cohort studies, case-control studies and other non-randomized intervention studies with an estimate of the relative effect of any occupational exposure to welding fumes on the prevalence of, incidence of or mortality from trachea, bronchus and lung cancer, compared with the theoretical minimum risk exposure level of no occupational exposure to welding fumes.

**Study appraisal and synthesis methods:**

At least two review authors will independently screen titles and abstracts against the eligibility criteria at a first stage and full texts of potentially eligible records at a second stage, followed by extraction of data from qualifying studies. Two or more review authors will assess risk of bias and the quality of evidence, using the Navigation Guide tool or approach. If feasible, we will combine relative risks using meta-analysis. We will report results using the preferred reporting items for systematic reviews and meta-analyses guidelines (PRISMA).

## Highlights

• WHO and ILO are developing joint estimates of the work-related burden of disease and injury.
• This is a protocol for a systematic review & meta-analysis to inform the estimates.
• The topic is occupational welding fumes exposure & trachea, bronchus & lung cancer.

## 1 Background

The World Health Organization (WHO) and the International Labour Organization (ILO) are developing their first joint estimates of the work-related burden of disease and injury (WHO/ILO Joint Estimates) (Ryder 2017). The organizations plan to estimate the numbers of deaths and disability-adjusted life years (DALYs) that are attributable to selected occupational risk factors. The WHO/ILO Joint Estimates will be based on already existing WHO and ILO methodologies for estimating the burden of disease for selected occupational risk factors (International Labour Organization, 2014, Pruss-Ustun, 2017). It will expand existing methodologies with estimation of the burden of several prioritized additional pairs of occupational risk factors and health outcomes. For this purpose, population attributable fractions, the proportional reduction in burden from the health outcome achieved by a reduction of exposure to the theoretical minimum risk exposure level (Murray et al. 2004), will be calculated for each additional risk factor-outcome pair. These fractions will be applied to the total disease burden envelopes for the health outcome from the WHO Global Health Estimates (World Health Organization, 2017).

The WHO/ILO Joint Estimates may include a methodology for estimating the burden of trachea, bronchus and lung cancer from occupational exposure to welding fumes if feasible, as one of the additional prioritized risk factor outcome pairs. To optimize parameters used in estimation models, WHO and ILO are conducting a systematic review and meta-analysis of studies that include estimates of the effect of occupational exposure to welding fumes on trachea, bronchus and lung cancer. In this article, we present the protocol for this systematic review, in parallel to presenting systematic review protocols or completed systematic reviews on other additional risk factor-outcome pairs elsewhere (Descatha et al., 2018, Descatha et al., 2020, Godderis et al., 2018, Hulshof et al., 2019, Li et al., 2018, Li et al., 2020, Mandrioli et al., 2018, Paulo et al., 2019, Rugulies et al., 2019, Teixeira et al., 2019, Tenkate et al., 2019). The WHO/ILO joint estimation methodology and the WHO/ILO Joint Estimates are separate from these systematic reviews; they will be described and reported elsewhere.

### 1.1 Rationale

To consider the feasibility of estimating the burden of trachea, bronchus and lung cancer from occupational exposure to welding fumes and to ensure that potential estimates of burden of disease are reported in adherence with the guidelines for accurate and transparent health estimates reporting (GATHER) (Stevens et al. 2016), WHO and ILO require a systematic review and meta-analysis of studies with estimates of the relative effect of any occupational exposure to welding fumes on the prevalence of, incidence of or mortality from trachea, bronchus and lung cancer, compared with the theoretical minimum risk exposure level of no occupational exposure to welding fumes. The theoretical minimum risk exposure level is the exposure level that would result in the lowest possible population risk, even if it is not feasible to attain this exposure level in practice (Murray et al. 2004).

In 2017, the International Agency for Research on Cancer (IARC) classified welding fumes as “carcinogenic to humans” (Guha et al., 2017, International Agency for Research on Cancer, 2018). IARC based this assessment on “sufficient evidence” from the more than 50 epidemiologic studies on the effect of exposure to welding fumes (assessed indirectly through welding process or material, branch of industry, job title, job task, expert assessment or self-report) on lung cancer (International Agency for Research on Cancer 2018).

We are aware of four published meta-analyses reporting on the effect of welding fume exposure on development of lung cancer (Ambroise et al., 2006, Honaryar et al., 2019, Moulin, 1997, Sjogren et al., 1994). While these meta-analyses vary in eligibility criteria of included studies, all suggested an increased risk in the development of lung cancer.

The earliest meta-analysis, which only included studies that accounted for smoking and asbestos exposure, examined stainless steel welders (assessed indirectly by self-report by a worker, workplace manager or spouse) and the occurrence of lung cancer (Sjogren et al. 1994). The calculated pooled relative risk estimate in three case-referent (case-control) and two cohort studies included in the meta-analysis was 1.94 (95% CI 1.28–2.93). However, the authors did not test for or measure heterogeneity in the meta-analysis or asses the quality of the body of evidence.

A 2006 meta-analysis, an update of Moulin 1997, included population surveys, case–control studies, and industry-based cohort studies to assess the relationship between lung cancers and welding (Ambroise et al. 2006). Combined relative risks (CRR) values for the cohort studies were 1.29 (95% CI 1.19 – 1.40; χ^2^_=_ 20.6, P = 0.99) and for the case-control studies were 1.27 (95% CI 1.11–1.46; χ^2^_=_ 13.0, P = 0.60) when only studies without reporting bias were included in the analysis. No further assessment of the quality of the evidence was reported. The authors attempted to control for confounding due to smoking and when crude and adjusted relative risks were available, it appeared that no or only slight confounding due to smoking was detected.

The most recently published meta-analysis analysed the studies included in the IARC assessment (Honaryar et al. 2019). Summary estimates, adjusted for confounding by smoking and exposure to asbestos, stratified by study design suggest increased relative risks in development of lung cancer of 1.29 (95% CI 1.20 to 1.39; I^2^ = 26.4%) across 22 cohort studies; 1.87 (1.53 to 2.29; I^2^ = 44.1%) across 15 case-control studies; and 1.17 (1.04 to 1.38; I^2^ = 41.2%) for eight case-control studies. However, to our knowledge, no systematic review has been conducted of studies with estimates of the effect of *occupational* exposure to welding fumes on trachea, bronchus and lung cancer. We have not identified any systematic review protocol on the topic (PROSPERO – accessed May 14, 2020).

Different contexts may result in different exposures and effects of these exposures on the health outcome. Work in the informal economy, for example, may lead to different exposures and exposure effects than does work in the formal economy. The informal economy is defined as “all economic activities by workers and economic units that are – in law or in practice – not covered or insufficiently covered by formal arrangements”, but excluding “illicit activities, in particular the provision of services or the production, sale, possession or use of goods forbidden by law, including the illicit production and trafficking of drugs, the illicit manufacturing of and trafficking in firearms, trafficking in persons and money laundering, as defined in the relevant international treaties” (p. 4) (104th International Labour Conference 2015). Therefore, we will consider the formality of the economy studied as a key contextual factor in studies included in our systematic review.

Our systematic review and meta-analysis will differ from previous efforts in that it will:

• Be tailored to the needs of estimation of disease burden.
• Be based on a pre-published, peer-reviewed protocol (presented in this article).
• Include studies of working-age (≥15 years) workers in the formal and informal economy
• Include other non-randomized intervention studies including quasi-experimental, controlled before-after studies and interrupted time series studies.
• Undergo all stages of a systematic review as defined in the Navigation Guide systematic review framework (Woodruff and Sutton 2014), including assessments of the risk of bias, quality of evidence and, respectively, strength of evidence, with the Navigation Guide’s tools and approaches (Lam et al. 2016a).
• Include only *occupational* exposure to welding fumes (not all exposures including environmental ones).
• Include as an outcome trachea, bronchus and update the literature on development of lung cancer
• Includes published and unpublished studies (not just published ones).
• Include studies published up to 2020.

### 1.2 Description of the risk factor

The definition of the risk factor, the risk factor levels and its theoretical minimum risk exposure level are presented in Table 1. The risk factor is defined as having two levels: Any occupational exposure to welding fumes and no occupational exposure to welding fumes. Absence of any occupational exposure to welding fumes is assumed to be the theoretical minimum risk exposure level. However, since the theoretical minimum risk exposure level is usually set empirically based on the causal epidemiological evidence, we may modify the assumed level as evidence suggests. If several studies report exposure levels differing from the standard levels we define here, then, if possible, we will convert the reported levels to the standard levels and, if not possible, we will report analyses on these alternate exposure levels as supplementary information in the systematic review.

**Table 1:** Definitions of the risk factor, risk factor levels and the minimum risk exposure level.

**Concept**	**Definition**
Risk factor	Occupational exposure to welding fumes from welding any material by any welding process
Risk factor levels	1. Any occupational exposure to welding fumes 2. No occupational exposure to welding fumes
Theoretical minimum risk exposure level	No occupational exposure to welding fumes

### 1.3 Description of the outcome

The WHO Global Health Estimates group outcomes into standard burden of disease categories (World Health Organization, 2017), based on standard codes from the International Statistical Classification of Diseases and Related Health Problems, 10th Revision (ICD-10) (World Health Organization 2015). The relevant WHO Global Health Estimates category for our systematic review is: “II.A7. Trachea, bronchus and lung cancer (World Health Organization, 2017), and this category covers ICD-10 codes “C33 Malignant neoplasm of trachea” and “C34 Malignant neoplasm of bronchus and lung”. Our systematic review will cover the entire disease burden of the relevant WHO Global Health Estimates category.

### 1.4 How the risk factor may impact the outcome

Official health estimates of the burden of disease attributable to an occupational risk factor require a sufficient level of scientific consensus that the risk factor causes the disease or other specified outcome (Stevens et al. 2016). The abovementioned conclusion of the working group of individual experts convened by IARC in 2017 is the most recent scientific consensus that exposure to welding fumes is a sufficient cause of lung cancer in humans (Guha et al., 2017, International Agency for Research on Cancer, 2018). In IARC Monograph Volume 118, the working group concluded based on a synthesis of evidence streams of mechanistic, animal and human studies that “Welding fumes are carcinogenic to humans and cause cancer of the lung (Group 1)”; therefore, welding fumes are an established risk factor for human health. The IARC hazard identification however did not focus specifically on the effect of *occupational* exposure to welding fumes (as opposed to any exposures, including both occupational and environmental ones), but this is the focus of the current systematic review and meta-analysis.

Causal diagrams are useful tools in epidemiologic research and evidence synthesis because they provide transparent, graphical solutions for organizing the current state of knowledge about research topics (Rehfuess et al. 2013). Causal diagrams, such as directed acyclic graphs (Greenland et al. 1999) and logic models (Anderson et al. 2011), visually present complex relationships between variables and provide the framework for identifying study inclusion/exclusion criteria, guiding the literature search strategy, informing the variables for data extraction, and examining the factors that may contribute to differences between studies. The exposure and outcome of interest, as well as confounders (variables that are associated with both the exposure and outcome) and mediators (variables that may influence the exposure on the causal path to the outcome), are presented on a single diagram, with arrowheads showing the directionality in the relationships.

**Fig. 1:**
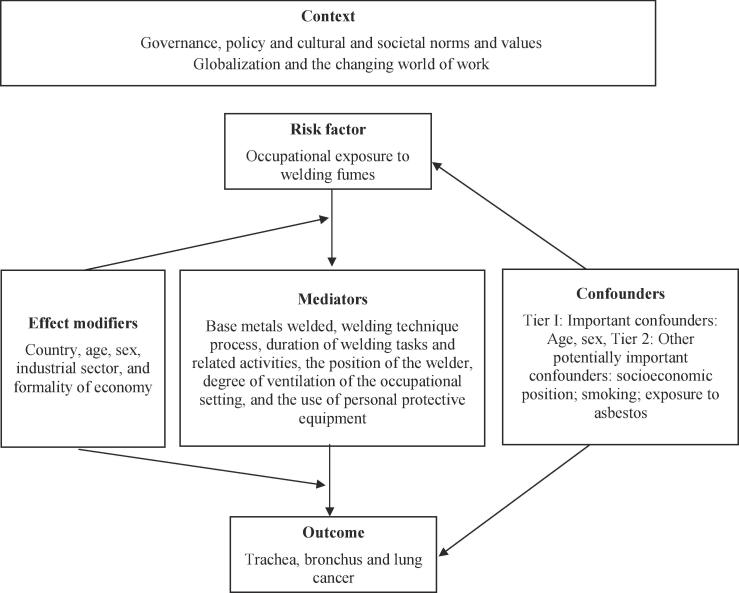
Logic model of the causal relationship between occupational exposure to welding fumes and trachea, bronchus and lung cancer.

Fig. 1 presents the logic model for our systematic reviews of the causal relationship between occupational exposure to welding fumes (risk factor) and trachea, bronchus and lung cancer (outcome). This is an *a priori*, process‐orientated logic model (Rehfuess et al. 2018) that seeks to capture the complexity of the risk factor-outcome causal relationship (Anderson et al. 2011). The Tier I: “Important confounders” are age and sex. The Tier 2: “Other potentially important confounders” are socioeconomic position, tobacco smoking and exposure to asbestos, which was commonly used as an insulating material in ships, the material covering rod electrodes, the cylinders holding acetylene gas, and the heat-protective equipment of welders and blankets to slow cooling of the weld (Fig. 1). Mediators are the factors that contributed to high variability in exposure to welding fumes: base metals welded, welding technique/process, duration of welding tasks and related activities (preparation, clean-up, breaks, etc.), the position of the welder, degree of ventilation of the occupational setting, and the use of personal protective equipment. Furthermore, the welders’ level of experience may also influence the particles generated from welding fumes (Chang et al. 2013); increased exposure may occur for apprentice welders or welders with minimal training (Graczyk et al. 2016).

## 2 Objectives

To systematically review and meta-analyse randomized control studies, cohort studies, case-control studies and other non-randomized intervention studies with estimates of the relative effect of occupational exposure to welding fumes on the prevalence of, incidence of or mortality from trachea, bronchus and lung cancer in any year among the working-age population, compared with the minimum risk exposure level of no exposure to welding fumes.

## 3 Methods

We will apply the Navigation Guide systematic review methodology for systematic reviews in environmental and occupational health as our guiding methodological framework (Woodruff and Sutton 2014), wherever feasible. The Navigation Guide applies established systematic review methods from clinical medicine, including standard Cochrane methods for systematic reviews of interventions, to the field of environmental and occupational health to ensure systematic and rigorous evidence synthesis on environmental and occupational risk factors that reduces bias and maximizes transparency (Woodruff and Sutton 2014). The need for further methodological development and refinement of the relatively novel Navigation Guide has been acknowledged (Woodruff and Sutton 2014). Our systematic review maps well to the Navigation Guide framework, and we will conduct steps 1–6 of this framework for the stream on human data, but not conduct any steps for the stream on non-human data, although we will briefly summarize narratively the evidence from non-human data that we are aware of.

This protocol adheres with the preferred reporting items for systematic review and meta-analysis protocols statement (PRISMA-P) (Shamseer et al., 2015). The abstract adhered with the reporting items for systematic reviews in journal and conference abstracts (PRISMA-A) (Belleret al. 2013). Any modification of the methods stated in the present protocol will be registered and reported in the systematic review itself. We will report the systematic review according to the preferred reporting items for systematic review and meta-analysis statement (PRISMA) (Liberatiet al. 2009). Our reporting of the parameters for estimating the burden of trachea, bronchus and lung cancer from occupational exposure to welding fumes in the systematic review will follow the GATHER guidelines (Stevens et al. 2016), because the WHO/ILO Joint Estimates that may be produced consecutive to the systematic review must also adhere to these reporting guidelines.

### 3.1 Eligibility criteria

The population, exposure, comparator and outcome (PECO) criteria (Morgan et al. 2018) are described below.

#### 3.1.1 Types of populations

We will include studies of working-age (≥15 years) workers in the formal and informal economy. Studies of children (aged < 15 years) and unpaid domestic workers will be excluded. Data on the formal and informal economy that the workers work in will be extracted. Participants residing in any WHO and/or ILO Member (or member) State and working in any industrial sector or occupation will be included. Occupational exposure to welding fumes may potentially have further population reach (e.g. as an environmental exposure, through the release of welding fumes from the workplace into the community); the scope of our systematic reviews will not be able to capture these populations and impacts on them. Appendix A in the Supplementary data provides a briefer overview of the PECO criteria.

#### 3.1.2 Types of exposures

We will include studies of occupational exposure to welding fumes in accordance with our standard definition (Table 1). Occupational exposure to welding fumes may be measured in several ways:

• Directly with quantitative measurement (e.g. by means of technology, such as air monitoring).
• Indirectly by proxy of occupation (or job title), such as by relevant codes and/or titles of the International Standard Classification of Occupations (ISCO) (International Labour Organization, 1966, International Labour Organization, 1987, International Labour Organization, 2012) (Table 2).

**Table 2:** International Standard Classification of Occupation (ISCO) codes and titles of occupations classified as exposed to welding fumes.

**ISCO revision**	**Code**	**Title**
ISCO-68 (International Labour Organization 1966)	87,200	Welders
	87,210	Gas & electric welders (general)
	87,215	Gas welders
	87,220	Electric arc welders (hand)
	87,225	Electric arc welders (machine)
	87,230	Thermite arc welders
	87,235	Resistance welders
ISCO-88 (International Labour Organization 1987)	7212	Welders and flame cutters
ISCO-08 (International Labour Organization 2012)	7212	Welders and flame cutters

Footnotes: ISCO-68 codes adopted from (Kendzia et al. 2013).
• Indirectly by job task of welding.
• Indirectly by classification in a job-exposure matrix (JEM) based on expert judgment or data external to the study.
• Indirectly by judgment of scientists with subject matter expertise.
• Indirectly by self-report by a worker or workplace manager, or by direct observation of the work process.

Studies using any of the preceding methods to identify occupational exposure to welding fumes will be eligible for inclusion. However, studies of workers whose jobs may include occasional or infrequent welding, such as plumbers, pipefitters or vehicle repairers, will be excluded from this review, but may be considered in a subsequent update. Studies using industrial sector as a proxy, which may be measured using the codes of the International Standard Industrial Classification of All Economic Activities (United Nations, 2008), will also be excluded, because we judged measurements of industrial sector to not be able to identify workers exposed to welding fumes. Similarly, studies that combine occupation as a welder into broad groups with other occupations or industrial sectors will not be eligible, as these groupings lack specificity for welding exposure (International Agency for Research on Cancer 2018).

If a study presents both direct and indirect measurements, and/or objective and subjective measurements, then we will prioritize direct and objective measurements. We will include studies with measures from any data source, including registry data.

#### 3.1.3 Types of comparators

The included comparator will be participants exposed to the theoretical minimum risk exposure level of no occupational exposure to welding fumes (Table 1). We will exclude all other comparators, including the general population.

#### 3.1.4 Types of outcomes

We will include studies that define trachea, bronchus and lung cancer in accordance with our standard definition of this outcome (see *1.3 Description of the outcome*). We will include studies that classify these cancers using the relevant diagnostic codes in ICD-10 (see above), ICD-9 (i.e., “162 Malignant neoplasm of trachea, bronchus, and lung”) or other versions of the ICD. Studies will also be included if they measure the outcome with methods that we judge to approximate the ICD-10 criteria (e.g. where an ICD code is not reported, it will be inferred from the information on the cancer site reported).

The following measurements of trachea, bronchus and lung cancer will be regarded as eligible:

i) Diagnosis by a physician with imaging.

ii) Hospital discharge records.

iii) Other relevant administrative data (e.g. records of sickness absence or disability).

iv) Registry data for diagnosis of and/or treatment for an eligible trachea, bronchus and lung cancer.

iv) Medically certified cause of death.

All other measures will be excluded from this systematic review.

Objective and subjective measures of the outcome will be eligible. If a study presents both objective and subjective measurements, then the objective ones will be selected.

#### 3.1.5 Types of studies

We will include studies that investigate the effect of occupational exposure to welding fumes on trachea, bronchus and lung cancer, for any study year or years, and over any period. Eligible study designs will be randomized controlled trials (including parallel-group, cluster, cross-over and factorial trials), cohort studies (both prospective and retrospective), case-control studies and other non-randomized intervention studies (including quasi-randomized controlled trials, controlled before-after studies and interrupted time series studies). We included a broader set of observational study designs than is commonly included, because a recent augmented Cochrane Review of complex interventions identified valuable additional studies using such a broader set of study designs (Arditi et al. 2016). As we have an interest in quantifying risk and not in qualitative assessment of hazard (Barroga and Kojima 2013), we will exclude all other study designs (e.g. uncontrolled before-and-after, cross-sectional, qualitative, modelling, case and non-original studies).

Records published in any year and any language will be included. The search will be conducted using English language terms, so that records published in any language that present essential information (i.e. title and abstract) in English will be included. If a record is written in a language other than those spoken by the authors of this review, then the record will be translated into English. Published and unpublished studies will be included. Studies conducted using unethical practices will be excluded (e.g., randomized controlled trials that deliberately exposed humans to a known risk factor to human health).

#### 3.1.6 Types of effect measures

We will include measures of the effect of any occupational exposure to welding fumes on the risk of having, developing or dying from cancer of the trachea, bronchus or lung, compared with the theoretical minimum risk exposure level (i.e., no such occupational exposure). Included are relative effect measures, namely risk ratios and odds ratios for prevalence measures, and hazard ratios for incidence measures (e.g., developed or died from a trachea, bronchus and lung cancer). Measures of absolute effects (e.g. mean differences in risks or odds) will be converted into relative effect measures, but if conversion is impossible, they will be excluded. To ensure comparability of effect estimates and facilitate meta-analysis, if a study presents an odds ratio, then we will convert it into a risk ratio, if possible, using the guidance provided in Cochrane’s handbook for systematic reviews of interventions (Deeks et al., 2019, Higgins and Green, 2011).

If a study presents estimates for the effect from two or more alternative models that have been adjusted for different variables, then we will systematically prioritize the estimate from the model that we consider best adjusted, applying the lists of confounders and mediators identified in our logic model (Fig. 1). We will generally prioritize estimates from models adjusted for more potential confounders over those from models adjusted for fewer. For example, if a study presents estimates from a crude, unadjusted model (Model A), a model adjusted for one potential confounder (Model B) and a model adjusted for two potential confounders (Model C), then we will prioritize the estimate from Model C. However, we will also consider the potential for over-adjustment in models that include non-confounders as covariates. We will prioritize estimates from models unadjusted for mediators over those from models that adjusted for mediators, because adjustment for mediators can introduce bias. For example, if Model A has been adjusted for two confounders and Model B has been adjusted for the same two confounders and a potential mediator, then we will choose the estimate from Model A. We prioritize estimates from models that can adjust for time-varying confounders that are at the same time also mediators, such as marginal structural models (Pega et al. 2016) over estimates from models that can only adjust for time-varying confounders, such as fixed-effects models (Gunasekara et al. 2014), over estimates from models that cannot adjust for time-varying confounding. If a study presents effect estimates from two or more potentially eligible models, we will explain why we prioritized the model we selected.

### 3.2 Information sources and search

#### 3.2.1 Electronic bibliographic databases

At a minimum, we (Martha S. Martínez-Silveira; MMS) will search the following electronic bibliographic databases:

1. International Clinical Trials Register Platform (inception to 30 April 2020).
2. CENTRAL (1 January 1996 to 30 April 2020).
3. Ovid Medline (1 January 1946 to 30 April 2020).
4. PubMed (1 January 1946 to 30 April 2020).
5. EMBASE (1 January 1947 to 30 April 2020).
6. Web of Science (1 January 1945 to 30 April 2020).
7. CISDOC (1 January 1901 to 31 December 2012).

The Ovid Medline search strategy is presented in Appendix B (see Supplementary data). To identify studies on trachea, bronchus and lung cancer, we adopted or adapted several search terms or strings used in a recent Cochrane Review on whole grain cereals for the primary or secondary prevention of trachea, bronchus and lung cancer (Kelly et al., 2017). We will perform searches in electronic databases operated in the English language using a search strategy in the English language. We will adapt the search syntax to suit the other electronic academic and grey literature databases. When we will be nearing completion of the review, we will update the PubMed database search for the most recent publications (e.g., e-publications ahead of print) over the last six months. Any deviation from the proposed search strategy in the actual search strategy will be documented and reported in the systematic review.

#### 3.2.2 Electronic grey literature databases

At a minimum, we (MSM) will search the two following electronic bibliographic databases:

1. OpenGrey (http://www.opengrey.eu/).
2. Grey Literature Report (http://greylit.org/).

#### 3.2.3 Internet search engines

We (MSM) will also search the Google (www.google.com/) and GoogleScholar (www.google.com/scholar/) Internet search engines and screen the first 100 hits for potentially relevant records, as has been done in Cochrane Reviews previously (Pega et al., 2015, Pega et al., 2017).

#### 3.2.4 Organizational websites

The websites of the following international organizations and national government departments will be searched by Seo Yeon Ahn (SYA), Alexis Descatha (AD), Angel Dzhambov (ADz), Neela Guha (NG), Seong-Kyu Kang (SKK), Alberto Modenese (AM), and Siyu Zhang (SZ):

1. International Labour Organization (www.ilo.org/).
2. World Health Organization (www.who.int).
3. International Agency for Research on Cancer (https://www.iarc.fr/)
4. European Agency for Safety and Health at Work (https://osha.europa.eu/en).
5. Eurostat (www.ec.europa.eu/eurostat/web/main/home).
6. China National Knowledge Infrastructure (http://www.cnki.net/).
7. Finnish Institute of Occupational Health (https://www.ttl.fi/en/).
8. United States National Institute of Occupational Safety and Health (NIOSH) of the United States of America, using the NIOSH data and statistics gateway (https://www.cdc.gov/niosh/data/).

#### 3.2.5 Hand-searching and expert consultation

We (SYA, AD, ADz, NG, SKK, and SZ) will hand-search for potentially eligible studies in:

• Reference list of previous systematic reviews.
• Reference list of all included study records.
• Study records published over the past 24 months in the three peer-reviewed academic journals with the largest number of included studies.
• Study records that have cited the included studies (identified in Web of Science citation database).
• Collections of the review authors.

Additional experts will be contacted with a list of included studies, with the request to identify potentially eligible additional studies.

### 3.3 Study selection

Study selection will be carried out with the Covidence software. All study records identified in the search will be downloaded and duplicates will be identified and deleted. Afterwards, at least two review authors (out of: SYA, AD, ADz, NG, AM, and SZ), working in pairs, will independently screen titles and abstracts (step 1) and then full texts (step 2) of potentially relevant records. A third review author (Dana Loomis; DL) will resolve any disagreements between the two review authors. Study selection may be supported by use of machine learning software, such as SWIFT. Study records will not be assigned to reviewers who have been authors of this study record. The study selection will be documented in a flow chart in the systematic review, as per PRISMA guidelines (Liberati et al. 2009).

### 3.4 Data extraction and data items

We will use the standard data extraction sheet that WHO and ILO have developed for their series of systematic reviews for the WHO/ILO Joint Estimates. The data extraction sheet will be trialled until data extractors reach convergence and agreement. At a minimum, two review authors (out of: SYA, AD, ADz, NG, SKK, AM, and SZ) will extract data on study characteristics (including study authors, study year, study country, participants, exposure and outcome), study design (including summary of study design, comparator, epidemiological models used and effect estimate measure), risk of bias (including selection bias, reporting bias, confounding and reverse causation) and study context (e.g. data on contemporaneous exposure to other occupational risk factors potentially relevant for deaths or other health loss from trachea, bronchus and lung cancer). A third review author (DL) will resolve conflicts in data extraction. Data will be entered into and managed with the Review Manager, Version 5.3 (RevMan 5.3) (2014) or DistillerSR (EvidencePartner 2017) software, but the Health Assessment Workspace Collaborative (HAWC) (Shapiro et al. 2018) may also be used in parallel or to prepare data for entry into RevMan 5.3.

Data from studies that were included in IARC Monograph 118 (International Agency for Research on Cancer 2018) and a subsequent meta-analysis (Honaryar et al. 2019) have already been extracted and are available to WHO in a database in the Table Builder software (Shapiro et al. 2018). Available items from this data extraction include: study authors, year, country, number of participants, exposure measures, and outcome measures. Extraction of these data was carried out by at least two data extractors, with comparisons of the database to the original sources. These existing data extractions will be used when they are available for studies determined to be eligible for inclusion in this review. However, ultimately, all data extraction items in the WHO/ILO standard data extraction sheet will be extracted for all included studies.

We will also extract data on potential conflict of interest in included studies. For each author and affiliated organization of each included study record, we will extract their financial disclosures and funding sources. We will use a modification of a previous method to identify and assess undisclosed financial interest of authors (Forsyth et al. 2014). Where no financial disclosure or conflict of interest statements are available, we will search the name of all authors in other study records gathered for this study and published in the prior 36 months and in other publicly available declarations of interests (Drazen et al., 2010a, Drazen et al., 2010b).

We will request missing data from the principal study author by email or phone, using the contact details provided in the principal study record. If we do not receive a response from the study author, we will send follow-up emails twice, at two and four weeks.

### 3.5 Risk of bias assessment

Standard risk of bias tools do not exist for systematic reviews of effects of exposure to occupational risk factors on health outcomes in occupational and environmental health (Pega et al., 2019). The five methods specifically developed for occupational and environmental health are for either or both hazard identification and risk assessment and they differ substantially in the types of studies (randomized, observational and/or simulation studies) and data (e.g. human, animal and/or in vitro) they seek to assess (Rooney et al. 2016). However, all five methods, including the Navigation Guide (Lam et al. 2016c), assess risk of bias in human studies similarly (Rooney et al. 2016).

The Navigation Guide was specifically developed to translate the rigor and transparency of systematic review methods applied in the clinical sciences to the evidence stream and decision context of environmental health (Woodruff and Sutton 2014), which includes workplace environment exposures and associated health outcomes. The Navigation Guide is our overall organizing framework and we will also apply its risk of bias assessment method in Systematic Review 2. The Navigation Guide risk of bias assessment method builds on the standard risk of bias assessment methods of Cochrane (Higgins et al. 2011) and the US Agency for Healthcare Research and Quality (Viswanathan et al. 2008). Some further refinements of the Navigation Guide method may be warranted (Goodman et al. 2017), but it has been successfully applied in several completed and ongoing systematic reviews (Johnson et al., 2016, Johnson et al., 2014, Koustas et al., 2014, Lam et al., 2016a, Lam et al., 2014, Lam et al., 2017, Vesterinen et al., 2014). In our application of the Navigation Guide method, we will draw heavily on one of its latest versions, as presented in the protocol for an ongoing systematic review (Lam et al. 2016c). Should a more suitable method become available, we may switch to it.

We will assess risk of bias on the individual study level and on the body of evidence overall. The nine risk of bias domains included in the Navigation Guide method for human studies are: (i) source population representation; (ii) blinding; (iii) exposure assessment; (iv) outcome assessment; (v) confounding; (vi) incomplete outcome data; (vii) selective outcome reporting; (viii) conflict of interest; and (ix) other sources of bias. While two of the earlier case studies of the Navigation Guide did not utilize outcome assessment as a risk of bias domain for studies of human data (Johnson et al., 2014, Koustas et al., 2014, Lam et al., 2014, Vesterinen et al., 2014), all of the subsequent reviews have included this domain (Johnson et al., 2016, Lam et al., 2016a, Lam et al., 2017, Lam et al., 2016b, Lam et al., 2016c). Risk of bias or confounding ratings will be: “low”; “probably low”; “probably high”; “high” or “not applicable” (Lam et al. 2016c). To judge the risk of bias in each domain, we will apply a priori instructions (Appendix C in the Supplementary data), which we have adopted or adapted from an ongoing Navigation Guide systematic review (Lam et al. 2016c).

All risk of bias assessors will jointly trial the application of the risk of bias criteria until they have synchronized their understanding and application of these criteria. At least two study authors (out of: SYA, Nicholas Chartres (NC), AD, ADz, NG, SKK, AM, Rebecca Morgan (RM), and SZ) will independently judge the risk of bias for each study for each domain by outcome. Where individual assessments differ, a third author (NC or RM) will resolve the conflict. In the systematic review, for each included study, we will report our study-level risk of bias assessments by domain (i.e. the selected rating and the justification for selecting this rating) in standard ‘Risk of bias’ tables (Higgins et al. 2011). For the entire body of evidence, we will present the study-level risk of bias assessment ratings by domain in a ‘Risk of bias summary’ figure. (Higgins et al. 2011).

### 3.6 Synthesis of results

We (ADz, NG, and DL) will conduct meta-analyses separately for estimates of the effect on prevalence, incidence and mortality. Studies of different designs will not be combined quantitatively. If we find two or more studies with an eligible effect estimate, two review authors (NG and DL) will independently investigate the clinical heterogeneity (Deeks et al. 2019) of the studies in terms of participants (including country, sex, age and industrial sector or occupation), level of risk factor exposure, comparator and outcomes. If we find that effect estimates differ considerably by country, sex and/or age, or a combination of these, then we will synthesise evidence separately by these factors or combination thereof. Differences by country could include or be expanded to include differences by country group (e.g. WHO region or World Bank income group). If we find that effect estimates are clinically homogenous across countries, sexes and age groups, then we will combine studies from all these populations into one pooled effect estimate that could be applied across all combinations of countries, sexes and age groups in the WHO/ILO Joint Estimates.

If we judge two or more studies for the relevant combination of country, sex and age group, or a combination thereof, to be sufficiently clinically homogenous to potentially be combined quantitatively using quantitative meta-analysis, then we will test the statistical heterogeneity of the studies using the I^2^ statistic (Figueroa 2014). If two or more clinically homogenous studies are found to be sufficiently homogenous statistically to be combined in a meta-analysis, we will pool the risk ratios of the studies in a quantitative meta-analysis, using the inverse variance method with a random effects model to account for cross-study heterogeneity (Figueroa 2014). The meta-analysis will be conducted in RevMan 5.3, but the data for entry into these programmes may be prepared using another recognized statistical analysis programme, such as Stata. We will neither quantitatively combine data from studies with different designs (e.g. combining cohort studies with case-controls studies), nor unadjusted and adjusted models. We will only combine studies that we judge to have a minimum acceptable level of adjustment for confounders (i.e. a study must contain at least one Tier I: Important confounders: Age or sex). In instances where two or more studies of the same data source (e.g. the same study cohort) are eligible for inclusion into the meta-analysis, we will prioritize in this order i) the study with the most informative assessment of exposure to welding fumes; ii) the study with the longest follow-up; iii) the study with the most complete control of relevant potential confounders. If our pre-specified rules for selecting a study’s result does not allow us to uniquely identify one for inclusion, we will randomly select one study. If quantitative synthesis is not feasible, we will synthesise the study findings narratively and identify the estimates that we judged to be the highest quality evidence available.

### 3.7 Additional analyses

If there is evidence for differences in effect estimates by country, sex, age, industrial sector and/or occupation, or by a combination of these variables, we (ADz, NG, and DL) will conduct subgroup analyses by the relevant variable or combination of variables, as feasible. Where both studies on workers in the informal economy and in the formal economy are included, we will conduct sub-group analyses by formality of economy. Findings of these subgroup analyses, if any, will be used as parameters for estimating burden of disease specifically for relevant populations defined by these variables. We will also conduct subgroup analyses by study design (e.g. randomized controlled trials versus cohort studies versus case-control studies).

At a minimum, we will perform a sensitivity analyses that will include only studies judged to be of “low” or “probably low” risk of bias from conflict of interest; judged to be of “low” or “probably low” risk of bias from confounding; judged to be of “low” or “probably low” risk of bias; with published data only; with studies that adjusted for smoking and asbestos exposure; and with documented or approximated ICD-10 diagnostic codes. We may also conduct a sensitivity analysis using an alternative meta-analytic model, namely the inverse variance heterogeneity (IVhet) model (Doi et al. 2017). We may also conduct a sensitivity dose–response meta-analysis of studies that report categorical risk estimates, which would enable us to investigate potential threshold effects (Xu and Doi 2017).

### 3.8 Quality of evidence assessment

Standard quality of evidence approaches do not exist for systematic reviews in occupational and environmental health, nor for risk assessment. We will assess quality of evidence using a modified version of the Navigation Guide quality of evidence assessment approach (Lam et al. 2016c). This is based on the GRADE approach (Schünemann et al., 2011), adapted specifically to systematic reviews in occupational and environmental health (Morgan et al. 2016). Should a more suitable method become available, we may switch to it.

All review authors will together judge quality of evidence for the entire body of evidence by outcome. We will adopt or adapt the latest Navigation Guide instructions (Appendix D in the Supplementary data) for grading the quality of evidence (Lam et al. 2016c). We will downgrade the quality of evidence for the following five GRADE reasons: (i) risk of bias; (ii) inconsistency; (iii) indirectness; (iv) imprecision; and (v) publication bias. If our systematic review includes ten or more studies, we will generate a funnel plot to ascertain presence of publication bias. If it includes nine or fewer studies, we will judge the risk of publication bias qualitatively.

We will grade the evidence, using the three Navigation Guide standard quality of evidence ratings: “high”, “moderate” and “low” (Lamet al. 2016c). Within each of the relevant domains, we will rate the concern for the quality of evidence, using the ratings “none”, “serious” and “very serious”. As per Navigation Guide, we will start at “high” for randomized studies and “moderate” for observational studies. Quality will be downgraded for no concern by nil grades (0), for a serious concern by one grade (-1) and for a very serious concern by two grades (-2). We will up-grade the quality of evidence for the following other reasons: large effect size, evidence of a dose–response relationship and plausibility that residual confounding and bias cannot explain the effect. For example, if we have a serious concern for risk of bias in a body of evidence consisting of observational studies (-1), but no other concerns and there are no reasons for upgrading, then we will downgrade its quality of evidence by one grade from “moderate” to “low”.

### 3.9 Strength of evidence assessment

We (all review authors) will apply the standard Navigation Guide methodology (Lam et al. 2016c) to rate the strength of the evidence. The rating will be based on a combination of four criteria: (i) quality of body of evidence; (ii) direction of effect; (iii) confidence in effect; and (iv) other compelling attributes of the data that may influence certainty. The ratings for strength of evidence for the effect of occupational exposure to welding fumes on trachea, bronchus and lung cancer will be “sufficient evidence of toxicity/harmfulness”, “limited of toxicity/harmfulness”, “inadequate of toxicity/harmfulness” and “evidence of lack of toxicity/harmfulness” (Appendix E in the Supplementary data for summary and definition of ratings).

## Financial support

FP is a salaried staff member of WHO. This publication was prepared with financial support to the 10.13039/100004423WHO from the National Institute for Occupational Safety and Health of the Centres for Disease Control and Prevention of the United States of America (Grant 1E11OH0010676-02; Grant 6NE11OH010461-02-01; and Grant 5NE11OH010461-03-00).

## Sponsors

The sponsors of this systematic review are the World Health Organization and the International Labour Organization.

## Author contributions

Had the idea for this systematic review: FP, Ivan Ivanov (WHO), Nancy Leppink (ILO).

Selected the lead reviewer and gathered the review teams: FP, Ivan Ivanov, Nancy Leppink, Yuka Ujita (ILO).

Coordinated the entire series of systematic reviews: FP, Yuka Ujita.

Is the lead reviewer of this systematic review: DL.

Led the design of the systematic review including developed the standard methods: FP.

Contributed substantially to the design of the systematic review: NC, NG, RM, DL.

Developed and piloted the search strategy: MMS.

Developed the standards and wrote the template for all protocols in the series: FP

Wrote the first draft of the protocol using the template: FP.

Revised the manuscript critically for important intellectual content: All authors.

Coordinated inputs from WHO, ILO and external experts: FP.

Ensured tailoring of the systematic review for WHO/ILO estimation purposes: FP.

Ensured harmonization across systematic reviews in the series: FP.

Approved the final version of the systematic review to be published: All authors.

Agreed to be accountable for all aspects of the work in ensuring that questions related to the accuracy or integrity of any part of the work are appropriately investigated and resolved: All authors.

Is the guarantor of the systematic review: DL.

## Declaration of Competing Interest

The authors declare that they have no known competing financial interests or personal relationships that could have appeared to influence the work reported in this paper.

## References

[b0005] Review Manager (RevMan). Version 5.3. Copenhagen: The Nordic Cochrane Centre, The Cochrane Collaboration; 2014.

[b0010] 104th International Labour Conference. Transition from the Informal to the Formal Economy (Recommendation No. 204). Geneva: International Labour Organization 2015.

[b0015] Ambroise D., Wild P., Moulin J.J. (2006). Update of a meta-analysis on lung cancer and welding. Scand J. Work Environ. Health.

[b0020] Anderson L.M., Petticrew M., Rehfuess E., Armstrong R., Ueffing E., Baker P., Francis D., Tugwell P. (2011). Using logic models to capture complexity in systematic reviews. Res. Synth. Methods.

[b0025] Arditi C., Burnand B., Peytremann-Bridevaux I. (2016). Adding non-randomised studies to a Cochrane review brings complementary information for healthcare stakeholders: an augmented systematic review and meta-analysis. BMC Health Serv. Res..

[b0030] Barroga E.F., Kojima T. (2013). Research study designs: an appraisal for peer reviewers and science editors. Eur. Sci. Ed..

[b0035] Beller, E.M., Glasziou, P.P., Altman, D.G., Hopewell, S., Bastian, H., Chalmers, I., Gotzsche, P.C., Lasserson, T., Tovey, D., Group, P.F.A., 2013. PRISMA for Abstracts: reporting systematic reviews in journal and conference abstracts. PLoS Med., 2013;10:e1001419. 10.1371/journal.pmed.1001419 PMC3621753 23585737

[b0040] Chang C., Demokritou P., Shafer M., Christiani D. (2013). Physicochemical and toxicological characteristics of welding fume derived particles generated from real time welding processes. Environ. Sci. Process Impacts.

[b0045] Deeks J., Higgins J., Altman D., Higgins J.P.T.T.J., Chandler J., Cumpston M., Li T., Page M.J., Welch V.A. (2019). Chapter 10: Analysing data and undertaking meta-analyses. Cochrane Handbook for Systematic Reviews of Interventions version 60.

[b0050] Descatha A., Sembajwe G., Baer M., Boccuni F., Di Tecco C., Duret C., Evanoff B.A., Gagliardi D., Ivanov I.D., Leppink N., Magnusson Hanson L.L., Marinaccio A., Ozguler A., Pega F., Pico F., Prüss-Üstün A.M., Ronchetti M., Roquelaure Y., Sabbath E., Stevens G.A., Tsutsumi A., Ujita Y., Iavicoli S. (2018). WHO/ILO work-related burden of disease and injury: Protocol for systematic reviews of exposure to long working hours and of the effect of exposure to long working hours on stroke. Environ. Int..

[b0055] Descatha A., Sembajwe G., Pega F., Ujita Y., Baer M., Boccuni F., Di Tecco C., Duret C., Evanoff B.A., Gagliardi D., Godderis L., Kang S.K., Kim B.J., Li J., Magnusson Hanson L.L., Marinaccio A., Ozguler A., Pachito D., Pell J., Pico F., Ronchetti M., Roquelaure Y., Rugulies R., Schouteden M., Siegrist J., Tsutsumi A., Iavicoli S. (2020). The effect of exposure to long working hours on stroke: A systematic review and meta-analysis from the WHO/ILO Joint Estimates of the Work-related Burden of Disease and Injury. Environ. Int..

[b0060] Doi S.A.R., Furuya-Kanamori L., Thalib L., Barendregt J.J. (2017). Meta-analysis in evidence-based healthcare: a paradigm shift away from random effects is overdue. Int. J. Evid. Based Healthc..

[b0065] Drazen J.M., de Leeuw P.W., Laine C., Mulrow C., DeAngelis C.D., Frizelle F.A., Godlee F., Haug C., Hebert P.C., James A., Kotzin S., Marusic A., Reyes H., Rosenberg J., Sahni P., Van der Weyden M.B., Zhaori G. (2010). Toward more uniform conflict disclosures: the updated ICMJE conflict of interest reporting form. JAMA.

[b0070] Drazen J.M., Van der Weyden M.B., Sahni P., Rosenberg J., Marusic A., Laine C., Kotzin S., Horton R., Hebert P.C., Haug C., Godlee F., Frizelle F.A., de Leeuw P.W., DeAngelis C.D. (2010). Uniform format for disclosure of competing interests in ICMJE journals. JAMA.

[b0075] EvidencePartner. DistillerSR. Accessed from: https://www.evidencepartners.com/products/distillersr-systematic-review-software/: EvidencePartner; 2017.

[b0080] Figueroa J.L. (2014). Distributional effects of Oportunidades on early child development. Soc. Sci. Med..

[b0085] Forsyth S.R., Odierna D.H., Krauth D., Bero L.A. (2014). Conflicts of interest and critiques of the use of systematic reviews in policymaking: an analysis of opinion articles. Syst. Rev..

[b0090] Godderis L., Bakusic J., Boonen E., Delvaux E., Ivanov I.D., Lambrechts M.-C., Latorraca C.O., Leppink N., Martimbianco A.L., Pega F., Prüss-Üstün A.M., Riera R., Ujita Y., Pachito D.V. (2018). WHO/ILO work-related burden of disease and injury: Protocol for systematic reviews of exposure to long working hours and of the effect of exposure to long working hours on alcohol use and alcohol use disorder. Environ. Int..

[b0095] Goodman J.E., Lynch H.N., Beck N.B. (2017). More clarity needed in the Navigation Guide systematic review framework. Environ. Int..

[b0100] Graczyk H., Lewinski N., Zhao J., Concha-Lozano N., Riediker M. (2016). Characterization of Tungsten Inert Gas (TIG) Welding Fume Generated by Apprentice Welders. Ann. Occup. Hyg..

[b0105] Greenland S., Pearl J., Robins J.M. (1999). Causal diagrams for epidemiologic research. Epidemiology.

[b0110] Guha N., Loomis D., Guyton K.Z., Grosse Y., El Ghissassi F., Bouvard V., Benbrahim-Tallaa L., Vilahur N., Muller K., Straif K. (2017). International Agency for Research on Cancer Monograph Working, G. Carcinogenicity of welding, molybdenum trioxide, and indium tin oxide. Lancet Oncol..

[b0115] Gunasekara F.I., Richardson K., Carter K., Blakely T. (2014). Fixed effects analysis of repeated measures data. Int. J. Epidemiol..

[b0120] Higgins, J., Altman, D., Sterne, J., Chapter 8: Assessing risk of bias in included studies. in: Higgins J., Green S., eds. Cochrane Handbook for Systematic Reviews of Interventions Version 510 [updated March 2011] The Cochrane Collaboration, 2011 Available from http://handbookcochraneorg; 2011.

[b0125] Higgins, J.; Green, S. Cochrane Handbook for Systematic Reviews of Interventions Version 5.1.0 [updated March 2011]. The Cochrane Collaboration, 2011. Available from http://handbook.cochrane.org. ed^eds; 2011.

[b0130] Honaryar M.K., Lunn R.M., Luce D., Ahrens W., t Mannetje A., Hansen J., Bouaoun L., Loomis D., Byrnes G., Vilahur N., Stayner L., Guha N. (2019). Welding fumes and lung cancer: a meta-analysis of case-control and cohort studies. Occup. Environ. Med..

[b0135] Hulshof C.T.J., Colosio C., Daams J.G., Ivanov I.D., Prakash K.C., Kuijer P., Leppink N., Mandic-Rajcevic S., Masci F., van der Molen H.F., Neupane S., Nygard C.H., Oakman J., Pega F., Proper K., Pruss-Ustun A.M., Ujita Y., Frings-Dresen M.H.W. (2019). WHO/ILO work-related burden of disease and injury: Protocol for systematic reviews of exposure to occupational ergonomic risk factors and of the effect of exposure to occupational ergonomic risk factors on osteoarthritis of hip or knee and selected other musculoskeletal diseases. Environ. Int..

[b0140] International Agency for Research on Cancer. IARC Monograph: Volume 118: Welding, Molybdenum Trioxide, and Indium Tin Oxide. Lyons, France: International Agency for Research on Cancer, 2018. 31268644

[b0145] International Labour Organization (1966). ISCO-68: International Standard Classification of Occupations.

[b0150] International Labour Organization (1987). ISCO–88: International Standard Classification of Occupations.

[b0155] International Labour Organization (2012). ISCO–08: International Standard Classification of Occupations.

[b0160] International Labour Organization (2014). Safety and health at work : a vision for sustainable prevention: XX World Congress on Safety and Health at Work 2014: Global Forum for Prevention, 24–27 August 2014, Frankfurt, Germany.

[b0165] Johnson P.I., Koustas E., Vesterinen H.M., Sutton P., Atchley D.S., Kim A.N., Campbell M., Donald J.M., Sen S., Bero L., Zeise L., Woodruff T.J. (2016). Application of the Navigation Guide systematic review methodology to the evidence for developmental and reproductive toxicity of triclosan. Environ. Int..

[b0170] Johnson P.I., Sutton P., Atchley D.S., Koustas E., Lam J., Sen S., Robinson K.A., Axelrad D.A., Woodruff T.J. (2014). The Navigation Guide - evidence-based medicine meets environmental health: systematic review of human evidence for PFOA effects on fetal growth. Environ. Health Perspect..

[b0175] Kelly S.A., Hartley L., Loveman E., Colquitt J.L., Jones H.M., Al-Khudairy L., Clar C., Germano R., Lunn H.R., Frost G., Rees K. (2017). Whole grain cereals for the primary or secondary prevention of cardiovascular disease. Cochrane Database Syst. Rev..

[b0180] Kendzia B., Behrens T., Jockel K.H., Siemiatycki J., Kromhout H., Vermeulen R., Peters S., Van Gelder R., Olsson A., Bruske I., Wichmann H.E., Stucker I., Guida F., Tardon A., Merletti F., Mirabelli D., Richiardi L., Pohlabeln H., Ahrens W., Landi M.T., Caporaso N., Consonni D., Zaridze D., Szeszenia-Dabrowska N., Lissowska J., Gustavsson P., Marcus M., Fabianova E., t Mannetje A., Pearce N., Tse L.A., Yu I.T., Rudnai P., Bencko V., Janout V., Mates D., Foretova L., Forastiere F., McLaughlin J., Demers P., Bueno-de-Mesquita B., Boffetta P., Schuz J., Straif K., Pesch B., Bruning T. (2013). Welding and lung cancer in a pooled analysis of case-control studies. Am. J. Epidemiol..

[b0185] Koustas E., Lam J., Sutton P., Johnson P.I., Atchley D.S., Sen S., Robinson K.A., Axelrad D.A., Woodruff T.J. (2014). The Navigation Guide - evidence-based medicine meets environmental health: systematic review of nonhuman evidence for PFOA effects on fetal growth. Environ. Health Perspect..

[b0190] Lam J., Koustas E., Sutton P., Cabana M., Whitaker E., Padula A., Vesterinen H., Daniels N., Woodruff T.J. (2016). Applying the Navigation Guide: Case Study #6.

[b0195] Lam J., Koustas E., Sutton P., Johnson P.I., Atchley D.S., Sen S., Robinson K.A., Axelrad D.A., Woodruff T.J. (2014). The Navigation Guide - evidence-based medicine meets environmental health: integration of animal and human evidence for PFOA effects on fetal growth. Environ. Health Perspect..

[b0200] Lam J., Lanphear B., Bellinger D., Axelrad D., McPartland J., Sutton P., Davidson L.I., Daniels N., Sen S., Woodruff T.J. (2017). Developmental PBDE exposure and IQ/ADHD in childhood: A systematic review and meta-analysis. Environ. Health Perspect..

[b0205] Lam, J., Sutton, P., Halladay, A., Davidson, L.I., Lawler, C., Newschaffer, C.J., Kalkbrenner, A., Joseph J. Zilber School of Public Health, Windham, G.C., Daniels, N., Sen, S., Woodruff, T.J., 2016b. Applying the Navigation Guide Systematic Review Methodology Case Study #4: Association between Developmental Exposures to Ambient Air Pollution and Autism. PLoS One 2016b;21.

[b0210] Lam J., Sutton P., Padula A.M., Cabana M.D., Koustas E., Vesterinen H.M., Whitaker E., Skalla L., Daniels N., Woodruff T.J. (2016). Applying the Navigation Guide Systematic Review Methodology Case Study #6: Association between Formaldehyde Exposure and Asthma: A Systematic Review of the Evidence: Protocol.

[b0215] Li J., Brisson C., Clays E., Ferrario M.M., Ivanov I.D., Landsbergis P., Leppink N., Pega F., Pikhart H., Prüss-Üstün A.M., Rugulies R., Schnall P.L., Stevens G.A., Tsutsumi A., Ujita Y., Siegrist J. (2018). WHO/ILO work-related burden of disease and injury: Protocol for systematic reviews of exposure to long working hours and of the effect of exposure to long working hours on ischaemic heart disease. Environ. Int..

[b0220] Li J., Pega F., Ujita Y., Brisson C., Clays E., Descatha A., Ferrario M.M., Godderis L., Iavicoli S., Landsbergis P.A., Metzendorf M.I., Morgan R.L., Pachito D.V., Pikhart H., Richter B., Roncaioli M., Rugulies R., Schnall P.L., Sembajwe G., Trudel X., Tsutsumi A., Woodruff T.J., Siegrist J. (2020). The effect of exposure to long working hours on ischaemic heart disease: A systematic review and meta-analysis from the WHO/ILO Joint Estimates of the Work-related Burden of Disease and Injury. Environ. Int..

[b0225] Liberati A., Altman D.G., Tetzlaff J., Mulrow C., Gotzsche P.C., Ioannidis J.P., Clarke M., Devereaux P.J., Kleijnen J., Moher D. (2009). The PRISMA statement for reporting systematic reviews and meta-analyses of studies that evaluate health care interventions: explanation and elaboration. PLoS Med..

[b0230] Mandrioli D., Schlünssen V., Adam B., Cohen R.A., Chen W., Colosio C., Fischer A., Godderis L., Göen T., Ivanov I.D., Leppink N., Mandic-Rajcevic S., Masci F., Nemery B., Pega F., Prüss-Üstün A.M., Sgargi D., Ujita Y., Van der Mierden S., Zungu M., Scheepers P. (2018). WHO/ILO work-related burden of disease and injury: Protocols for systematic reviews of occupational exposure to dusts and/or fibres and of the effect of occupational exposure to dusts and/or fibres on pneumoconiosis. Environ. Int..

[b0240] Morgan R.L., Thayer K.A., Bero L., Bruce N., Falck-Ytter Y., Ghersi D., Guyatt G., Hooijmans C., Langendam M., Mandrioli D., Mustafa R.A., Rehfuess E.A., Rooney A.A., Shea B., Silbergeld E.K., Sutton P., Wolfe M.S., Woodruff T.J., Verbeek J.H., Holloway A.C., Santesso N., Schunemann H.J. (2016). GRADE: Assessing the quality of evidence in environmental and occupational health. Environ. Int..

[b0245] Morgan R.L., Whaley P., Thayer K.A., Schunemann H.J. (2018). Identifying the PECO: A framework for formulating good questions to explore the association of environmental and other exposures with health outcomes. Environ. Int..

[b0250] Moulin J.J. (1997). A meta-analysis of epidemiologic studies of lung cancer in welders. Scand J. Work Environ. Health.

[b0255] Murray C.J.L., Ezzati M., Lopez A.D., Rodgers A., Vander Hoorn S., Ezzati M., Lopez A.D., Rodgers A., Murray C.J.L. (2004). Comparative Quantification of Health Risks: Conceptual Framework and Methodological Issues. Comparative Quantification of Health Risks: Global and Regional Burdedn of Disease Attributable to Selected Major Risk Factors.

[b0260] Paulo M.S., Adam B., Akagwu C., Akparibo I., Al-Rifai R.H., Bazrafshan S., Gobba F., Green A.C., Ivanov I., Kezic S., Leppink N., Loney T., Modenese A., Pega F., Peters C.E., Pruss-Ustun A.M., Tenkate T., Ujita Y., Wittlich M., John S.M. (2019). WHO/ILO work-related burden of disease and injury: Protocol for systematic reviews of occupational exposure to solar ultraviolet radiation and of the effect of occupational exposure to solar ultraviolet radiation on melanoma and non-melanoma skin cancer. Environ. Int..

[b0265] Pega F., Blakely T., Glymour M.M., Carter K.N., Kawachi I. (2016). Using Marginal Structural Modeling to Estimate the Cumulative Impact of an Unconditional Tax Credit on Self-Rated Health. Am. J. Epidemiol..

[b0275] Pega F., Liu S.Y., Walter S., Lhachimi S.K. (2015). Unconditional cash transfers for assistance in humanitarian disasters: effect on use of health services and health outcomes in low- and middle-income countries. Cochrane Database Syst. Rev..

[b0280] Pega F., Liu S.Y., Walter S., Pabayo R., Saith R., Lhachimi S.K. (2017). Unconditional cash transfers for reducing poverty and vulnerabilities: effect on use of health services and health outcomes in low- and middle-income countries. Cochrane Database Syst. Rev..

[b0285] Pega F., Norris S.L., Backes C., Bero L.A., Descatha A., Gagliardi D., Godderis L., Loney T., Modenese A., Morgan R.L., Pachito D., Paulo M.B.S., Scheepers P.T.J., Schlunssen V., Sgargi D., Silbergeld E.K., Sorensen K., Sutton P., Tenkate T., Correa Torreao, da Silva D., Ujita Y., van Deventer E., Woodruff T.J., Mandrioli D. RoB-SPEO (2019). A tool for assessing risk of bias in studies estimating the prevalence of exposure to occupational risk factors from the WHO/ILO Joint Estimates of the Work-related Burden of Disease and Injury. Environ. Int..

[b0290] Pruss-Ustun, A., Wolf, J., Corvalan, C., Bos, R., Neira, M., 2017. Preventing disease through healthy environments: a global assessment of the burden of disease from enviornmental risks in: Department of Public Health E.a.S.D.o.H., ed. Geneva: World Health Organization.

[b0295] Rehfuess E.A., Best N., Briggs D.J., Joffe M. (2013). Diagram-based Analysis of Causal Systems (DACS): elucidating inter-relationships between determinants of acute lower respiratory infections among children in sub-Saharan Africa. Emerg. Themes Epidemiol..

[b0300] Rehfuess E.A., Booth A., Brereton L., Burns J., Gerhardus A., Mozygemba K., Oortwijn W., Pfadenhauer L.M., Tummers M., van der Wilt G.J., Rohwer A. (2018). Towards a taxonomy of logic models in systematic reviews and health technology assessments: A priori, staged, and iterative approaches. Res. Synth. Methods.

[b0305] Rooney A.A., Cooper G.S., Jahnke G.D., Lam J., Morgan R.L., Boyles A.L., Ratcliffe J.M., Kraft A.D., Schunemann H.J., Schwingl P., Walker T.D., Thayer K.A., Lunn R.M. (2016). How credible are the study results? Evaluating and applying internal validity tools to literature-based assessments of environmental health hazards. Environ. Int..

[b0310] Rugulies R., Ando E., Ayuso-Mateos J.L., Bonafede M., Cabello M., Di Tecco C., Dragano N., Durand-Moreau Q., Eguchi H., Gao J., Garde A.H., Iavicoli S., Ivanov I.D., Leppink N., Madsen I.E.H., Pega F., Pruss-Ustun A.M., Rondinone B.M., Sorensen K., Tsuno K., Ujita Y., Zadow A. (2019). WHO/ILO work-related burden of disease and injury: Protocol for systematic reviews of exposure to long working hours and of the effect of exposure to long working hours on depression. Environ. Int..

[b0315] Ryder G. (2017). Welcome address from the Director General of the International Labour Organization. XXI World Congress on Safety and Health at Work. Sands Expo and Convention Centre, Singapore.

[b0320] Schünemann, H., Oxman, A., Vist, G., Higgins, J., Deeks, J., Glasziou, P., Guyatt, G., 2011. Chapter 12: Interpreting results and drawing conclusions. In: Higgins, J., Green, S. (Eds.), Cochrane Handbook for Systematic Reviews of Interventions Version 510 [updated March 2011]. Available from www.handbook.cochrane.org: The Cochrane Collaboration.

[b0325] Shamseer L., Moher D., Clarke M., Ghersi D., Liberati A., Petticrew M., Shekelle P., Stewart L.A., Group, P.-P (2015). Preferred reporting items for systematic review and meta-analysis protocols (PRISMA-P) 2015: elaboration and explanation. BMJ.

[b0330] Shapiro A.J., Antoni S., Guyton K.Z., Lunn R.M., Loomis D., Rusyn I., Jahnke G.D., Schwingl P.J., Mehta S.S., Addington J., Guha N. (2018). Software Tools to Facilitate Systematic Review Used for Cancer Hazard Identification. Environ. Health Perspect..

[b0335] Sjogren B., Hansen K.S., Kjuus H., Persson P.G. (1994). Exposure to stainless steel welding fumes and lung cancer: a meta-analysis. Occup. Environ. Med..

[b0340] Stevens G.A., Alkema L., Black R.E., Boerma J.T., Collins G.S., Ezzati M., Grove J.T., Hogan D.R., Hogan M.C., Horton R., Lawn J.E., Marusic A., Mathers C.D., Murray C.J., Rudan I., Salomon J.A., Simpson P.J., Vos T., Welch V. (2016). Guidelines for Accurate and Transparent Health Estimates Reporting: the GATHER statement. Lancet.

[b0345] Teixeira, L.R., Azevedo, T.M., Bortkiewicz, A., Correa da Silva, D.T., de Abreu, W., de Almeida, M.S., de Araujo, M.A.N., Gadzicka, E., Ivanov, I.D., Leppink, N., Macedo, M.R.V., de, S.M.E.M.G., Pawlaczyk-Luszczynska, M., Pega, F., Pruss-Ustun, A.M., Siedlecka, J., Stevens, G.A., Ujita, Y., Braga, J.U., 2019. WHO/ILO work-related burden of disease and injury: Protocol for systematic reviews of exposure to occupational noise and of the effect of exposure to occupational noise on cardiovascular disease. Environ. Int., 125, 567–578. 10.1016/j.envint.2018.09.040 30683322

[b0350] Tenkate T., Adam B., Al-Rifai R.H., Chou B.R., Gobba F., Ivanov I.D., Leppink N., Loney T., Pega F., Peters C.E., Pruss-Ustun A.M., Silva Paulo M., Ujita Y., Wittlich M., Modenese A. (2019). WHO/ILO work-related burden of disease and injury: Protocol for systematic reviews of occupational exposure to solar ultraviolet radiation and of the effect of occupational exposure to solar ultraviolet radiation on cataract. Environ. Int..

[b0355] United Nations. ISIC Rev. 4: International Standard Industrial Classification of All Economic Activities, Revision 4. Statistical Papers Series M No. 4/Rev.4. in: Affairs D.o.E.a.S., ed. New York, NY: United Nations; 2008.

[b0360] Vesterinen H., Johnson P., Atchley D., Sutton P., Lam J., Zlatnik M., Sen S., Woodruff T. (2014). The relationship between fetal growth and maternal glomerular filtration rate: a systematic review. J. Maternal Fetal Neonatal Med..

[b0365] Viswanathan M., Ansari M.T., Berkman N.D., Chang S., Hartling L., McPheeters M., Santaguida P.L., Shamliyan T., Singh K., Tsertsvadze A., Treadwell J.R. (2008). Assessing the Risk of Bias of Individual Studies in Systematic Reviews of Health Care Interventions.

[b0370] Woodruff T.J., Sutton P. (2014). The Navigation Guide systematic review methodology: a rigorous and transparent method for translating environmental health science into better health outcomes. Environ. Health Perspect..

[b0375] World Health Organization (2015). ICD-10: International Statistical Classification of Diseases and Related Health Problems: 10th Revision.

[b0380] World Health Organization. WHO methods and data sources for global burden of disease estimates 2000-2015. Global Health Estimates Technical Paper WHO/HIS/IER/GHE/2017.1. In: Department of Information E.a.R., ed. Geneva: World Health Organization; 2017.

[b0385] Xu C., Doi S.A.R. (2017). The robust error meta-regression method for dose-response meta-analysis. Int J Evid Based Healthc.

